# Nanostructured Copper-Based Electrodes Electrochemically Synthesized on a Carbonaceous Gas Diffusion Membrane with Catalytic Activity for the Electroreduction of CO_2_

**DOI:** 10.1021/acsami.1c18844

**Published:** 2021-11-26

**Authors:** Martina Serafini, Federica Mariani, Andrea Fasolini, Erika Scavetta, Francesco Basile, Domenica Tonelli

**Affiliations:** Department of Industrial Chemistry “Toso Montanari”, University of Bologna, Viale del Risorgimento, 4, 40136 Bologna, Italy

**Keywords:** nanostructure, Cu-based electrocatalyst, CO_2_ conversion, electroreduction, acetic acid, gas diffusion layer

## Abstract

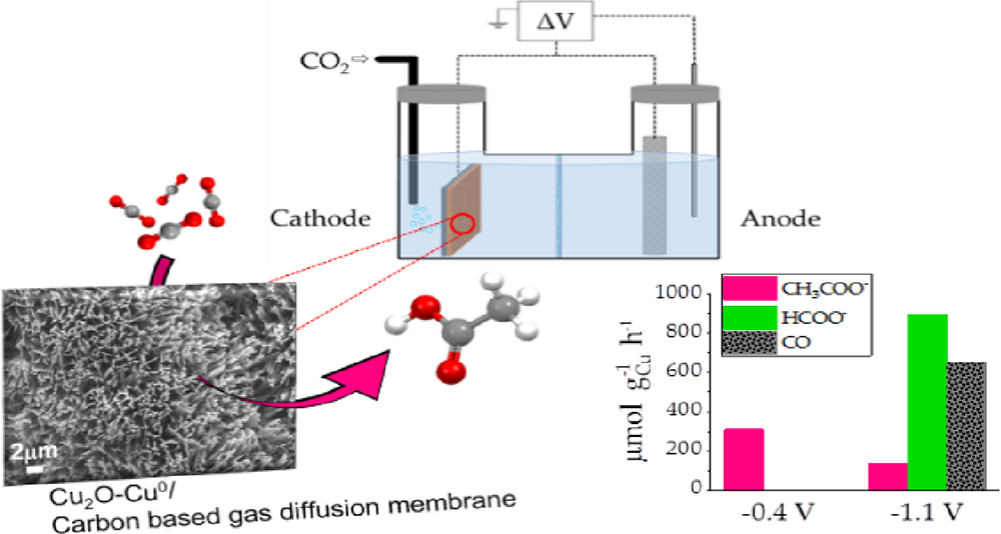

In this work, four different 4 cm^2^-sized nanostructured Cu-based electrocatalysts have been designed by a one-step electrodeposition process of Cu metal on a three-dimensional carbonaceous membrane. One consisted of Cu^0^, and the other three were obtained by further simple oxidative treatments. Morphological, structural, and electrochemical investigations on the four materials were carried out by scanning electron microscopy, Raman spectroscopy, X-ray diffraction, linear sweep voltammetry, and potential-controlled electrolysis. All the electrocatalysts showed promising catalytic activities toward CO_2_ electroreduction in liquid phase, with a remarkable selectivity toward acetic acid achieved when using the oxidized materials. In particular, the best electrocatalytic activity was observed for the Cu_2_O-Cu^0^ catalyst, working at a relatively low potential (−0.4 V vs RHE), which exhibited a stable and low current density of 0.46 mA cm^–2^ and a productivity of 308 μmol g_cat_^–1^ h^–1^. These results were attributed to the nanostructured morphology that is characterized by many void spaces and by a high surface area, which should guarantee a large number of Cu^I^ and Cu^0^ catalytic active sites. Moreover, kinetic analyses and preliminary studies about catalyst regeneration highlighted the stability of the best-performing catalyst.

## 1 Introduction

The global effort in pursuing effective decarbonization strategies is depicting new scenarios toward alternative production chains. In particular, CO_2_ capture and utilization play a key role in view of a substantial technological revolution in the way that energy is produced, stored, and converted nowadays from and among different energy sources and vectors. The electrochemical reduction of CO_2_, under liquid- or gas-phase conditions, is a reaction by which hydrocarbons can be obtained at ambient temperature and atmospheric pressure, thus closing the carbon cycle with the production of added-value commodity chemicals, using electricity derived preferably from renewables. In this context, the fascinating concept of solar-driven chemistry, realized with photochemical systems either including inorganic^1−7^ or organic biohybrid^8^ components, is attracting much attention thanks to the biomimetic approach toward a sustainable process, which culminates in the concept of artificial leaves.^2,9,10^ In such a system, light is absorbed at a photoanode to generate electricity that is used in situ for the electroreduction of CO_2_ to chemicals, thus ideally eliminating the need for any external power supply. Of particular interest is the direct production of methanol or acetic acid, which are otherwise obtained in a high-temperature, multistep process from methane-derived syngas (MeOH) and successive carbonylation (CH_3_COOH).^4^ To date, the design of lightweight, low-cost, and low-power consuming electrocatalytic platforms for high-throughput CO_2_ electrocatalytic reduction (CO_2_ER) represents a major challenge for future integration in the aforementioned artificial leaf. In this context, this work aims to develop novel active materials for the CO_2_ER.^11^ Due to its molecular structure, CO_2_ is particularly stable from a thermodynamic point of view, and its chemical transformation into products generally requires harsh temperature and pressure conditions. Conversely, a high overpotential is required for carbon dioxide electroreduction,^11^ which generally suffers from sluggish kinetics, multiphase rate-limiting steps, and poor selectivity, as well as intermediate-sharing, multiple reaction pathways, whose mechanisms are still under debate. On the other hand, experimental parameters such as the catalyst properties, local pH, chemical nature of the electrolyte solution, and applied potential dramatically affect the reaction and, consequently, the product distribution.^1,12,13^

Due to its unique ability to catalyze CO_2_ER toward a number of hydrocarbons, aldehydes, and alcohols requiring more than two electron transfers with substantial Faradaic efficiencies (FE), Cu has been overall recognized as the gold standard among pure metal catalysts since 1985.^13−15^

In particular, Cu-based electrode interfaces are able to stabilize the chemisorbed CO_2_^·^^–^ radical anions and CO species, which are the key intermediates in the initial phase of the catalytic CO_2_ conversion and in the subsequent process of hydrocarbon and alcohol formation, respectively. This not only increases the CO surface concentration, thereby promoting those CO_2_ER pathways involving C–C coupling reactions, but also blocks the reaction sites for the parasitic hydrogen evolution reaction (HER).^16,17^ Recent studies concerning liquid-phase CO_2_ER have reported on the fundamental role of nano- and microstructures,^18−21^ as well as the redox state^22,23^ of Cu-based cathode materials in improving the catalyst’s selectivity and activity.

It has been shown that metallic Cu obtained after reduction of Cu_2_O, which was formed by electropolishing and subsequent thermal annealing of a polycrystalline Cu foil, displayed enhanced current densities and Faradaic efficiencies with respect to the pristine Cu foil.^24^ The more active Cu catalyst was produced in situ during CO_2_ER at −0.5 V vs RHE and led to formate production. The effect of the orientation and thickness of Cu_2_O films electrodeposited on commercial Cu plates for CO_2_ER performed at −1.1 V vs RHE was investigated by online electrochemical mass spectroscopy and XRD.^22^ On the one hand, the selectivity toward the main product (formic acid) was demonstrated to largely depend on the film thickness rather than the orientation. On the other hand, the reaction was found to be actually catalyzed by in situ formation of nanostructured Cu domains during the progress of the reaction itself. The influence of the CuO nanoparticle (NP) morphology synthesized by a hydrothermal method was studied during CO_2_ER at −1.7 V vs RHE.^25^ Again, in situ reduction to metallic Cu was reported, leading to good selectivity toward ethanol, while different electrocatalytic activities were observed depending on the initial CuO NP morphologies. Dendritic Cu catalysts electrodeposited on technical Cu mesh supports were also studied for CO_2_ electroreduction showing good Faradaic selectivity to formate and ethylene at −0.7 and −1.1 V vs RHE, respectively.^17^ Subsequent thermal annealing led to C_1_ > alcohol formation (EtOH and *n*-PrOH at −1.0 V vs RHE).

Thermal and electrochemical activations of copper foils to form oxide-based catalysts for CO_2_ER have been recently compared by Giri et al.^26^ Interestingly, the authors observed no relevant differences between the oxidation methods and showed, by XPS and XRD analyses, that all oxides reduced back to Cu^0^ within the first few minutes of the reaction. Higher currents and Faradaic efficiencies were obtained from the activated samples and were ascribed to the increased surface area after Cu activation. Overall, these works suggest that the CO_2_ER actually proceeds on Cu^0^ sites that form during the first stages of the reaction. Nevertheless, the redox state and morphology of the starting oxides affect the reaction outcome in terms of selectivity and the chemical nature of the products.

In other recent studies, the morphological evolution of electrochemically deposited cuprous oxide nanocubes during CO_2_ER has been monitored using liquid cell transmission electron microscopy^27^ (in situ TEM). Interestingly, pulsed electrolysis conditions were chosen to continuously regenerate Cu(I) species, and the combination of Cu(100) domains, defect sites, and surface Cu_2_O was found to enhance the CO_2_ER reaction pathway leading to C_2+_ products.^23^ Similarly, a high C_2_ Faradaic efficiency (80%) was reported toward acetic acid and ethanol using a 3D dendritic Cu/Cu_2_O composite electrochemically synthesized on a Cu foil, upon application of a reduction potential as low as −0.4 V vs RHE.^28^

Efforts in the replacement of bulk metal cathodes with lightweight and low-cost carbonaceous supports, especially large-area gas diffusion electrodes, represent a step forward in the overall design of artificial photosynthesis-oriented systems. The first attempts involving Cu-based catalysts for CO_2_ER date back to the 90s and were motivated by the increased mass fixation of CO_2_ provided by the porous and 3D structure of gas diffusion layers (GDLs).^29,30^ In more recent years, only a few works on liquid-phase CO_2_ER catalysts based on Cu^0^ NPs, either alone^31^ or deposited on CNTs,^4,32,33^ and loaded on GDLs have been reported, where formic and acetic acids were the major products. However, to date the role of the Cu morphology and redox state on CO_2_ER product distribution has not yet been investigated on GDL supports, which is a crucial step to pave the way for the use of such low-cost and high-surface area supports in the challenging electrochemical reaction.

In this work, a set of nanostructured Cu catalysts have been loaded over a carbonaceous GDL (Toray carbon paper, shortly named CP) by simple and highly reproducible procedures, including first the Cu^0^ electrodeposition and then a chemical or electrochemical oxidative treatment, leading to the formation of a pristine Cu^0^/CP and its oxidized forms Cu_2_O-Cu^0^/CP, CuO_*x*_-Cu^0^/CP, and Cu(OH)_2_-Cu^0^/CP.

The materials have been exploited for CO_2_ER in liquid phase to produce acetic acid as the main product. The catalytic performances in terms of productivity and selectivity toward formic and acetic acids depend on the electrocatalyst morphology and the Cu redox state, which can be finely tuned modifying the experimental parameters of the oxidative treatment.

## 2 Materials

### 2.1 Chemicals and Materials

Toray carbon paper (TGP-H-60), Nafion membrane N-115 (0.125 mm-thick, ≥0.90 meq/g exchange capacity), copper tape, and ammonium nitrate were purchased from Alfa Aesar. Copper nitrate trihydrate (Cu(NO_3_)_2_·3H_2_O), sulfuric acid, ethanol (96.0–97.2%), potassium hydroxide, hydrogen peroxide, ammonium persulfate, potassium hydrogen carbonate, and phenol were purchased from Sigma-Aldrich. Pure carbon dioxide (≥99.9%) was acquired from Rivoira S.r.l. Deuterium oxide (99.96%) was purchased from Eurisotop. Gas sampling bags (Tedlar bags) were obtained from Supelco. All chemicals were of reagent grade or higher.

### 2.2 Apparatus

The copper electrodeposition and the electrochemical characterizations of the support were carried out in a conventional three-electrode cell, and all potentials were controlled by a potentiostat (CH Instrument 660 C). Toray carbon paper (4 cm^2^-sized) was used as the working electrode, while a saturated calomel electrode (SCE) and a Pt gauze were used as the reference and counter electrodes, respectively. With the exception of the electrocatalytic tests, all the potentials were quoted vs SCE. The morphology and the structure of the catalysts were investigated by scanning electron microscopy (SEM) using an E-SEM Zeiss EVO 50 series instrument. Energy-dispersive X-ray spectroscopy (EDS) measurements were performed with an Oxford INCA system equipped with a 30 mm^2^ silicon drift detector. Raman spectra were recorded with a Renishaw Raman RM1000 equipped with a Leica DMLM optical microscope. The excitation wavelength came from an argon laser adjusted at 514.5 nm with an outpower power of 25 mW. This power was reduced as needed by neutral density filters in order to prevent sample damage. The crystalline phases and the dimensions of the crystallites were analyzed by X-ray diffraction spectroscopy (XRD) using a PW1050/81 diffractometer (Philips/Malvern, Royston, UK) coupled with a graphite monochromator in the diffracted beam and controlled by a PW1710 unit (Cu Kα, λ = 0.15418 nm). The electrochemical CO_2_ reduction reaction tests were carried out in a low-volume, two-compartment electrochemical (H-type) cell (Pine Reasearch Instrumentation, Inc.). The GDLs coated with the catalysts were used as the working electrodes, and a Ag/AgCl (KCl sat.) electrode and a Pt gauze were used as the reference and counter electrodes, respectively. ^1^H NMR spectra were recorded by means of an Inova 600 spectrometer (600 MHz, D_2_O) coupled with a triple resonance probe. Gaseous products were detected by a Thermo Focus GC with a carbon molecular sieve column (CARBOSPHERE 80/100 6×1/8) equipped with a TCD detector.

### 2.3 Pretreatment of the Carbonaceous Support

A total geometrical surface area of 4 cm^2^ of Toray carbon paper (CP) was obtained from a 19 × 19 cm foil. Before copper deposition, the carbonaceous membrane was soaked in 1 M H_2_SO_4_ followed by treatment in pure EtOH for well-defined times, whose optimization will be discussed in Section 3.1.2.

### 2.4 Electrodeposition of Cu^0^ on the Carbon Paper Electrode

The electrodeposition of metal copper was performed in a conventional three-electrode cell by applying a constant potential of −0.4 V vs SCE for 500 s. The electrolytic solution was composed of 0.15 M Cu(NO_3_)_2_·3H_2_O and 0.70 M NH_4_NO_3_. The pretreated CP was employed as the working electrode and coupled with a Pt gauze and the SCE as counter and reference electrodes, respectively. The as-obtained Cu^0^ thin layer was carefully washed in monodistilled H_2_O and then dried at 333 K overnight. The pristine metal copper electrocatalyst was named Cu^0^/CP.

### 2.5 Oxidative Treatment of the Pristine Cu^0^/CP

Once the Cu^0^ electrocatalyst had been obtained, the copper oxidation state was tuned by means of simple chemical and electrochemical methods. Three different oxidized catalysts were obtained as follows.

(a) The Cu_2_O-Cu^0^/CP catalyst was achieved by applying a constant potential of −0.4 V vs SCE for 720 s in 0.5 M KOH aqueous solution, obtaining a thin Cu_2_O film over the metal copper deposit.
(b) The Cu(OH)_2_-Cu^0^/CP and the CuO_*x*_-Cu^0^/CP catalysts were chemically obtained. Indeed, Cu^0^/CP electrodes were soaked for 20 min in two different 2.5 M NaOH solutions adding either 0.1 M (NH_4_)_2_S_2_O_8_ in order to obtain the copper hydroxide or 0.1 M H_2_O_2_ to obtain a mixture of copper oxides, along with a metal copper layer that, in both cases, was still present.

The morphologies and the structures of each electrocatalyst were thoroughly investigated by SEM-EDS, Raman, and X-ray diffraction analyses.

### 2.6 Liquid-Phase CO_2_ Electroreduction Tests

**Figure 1:**
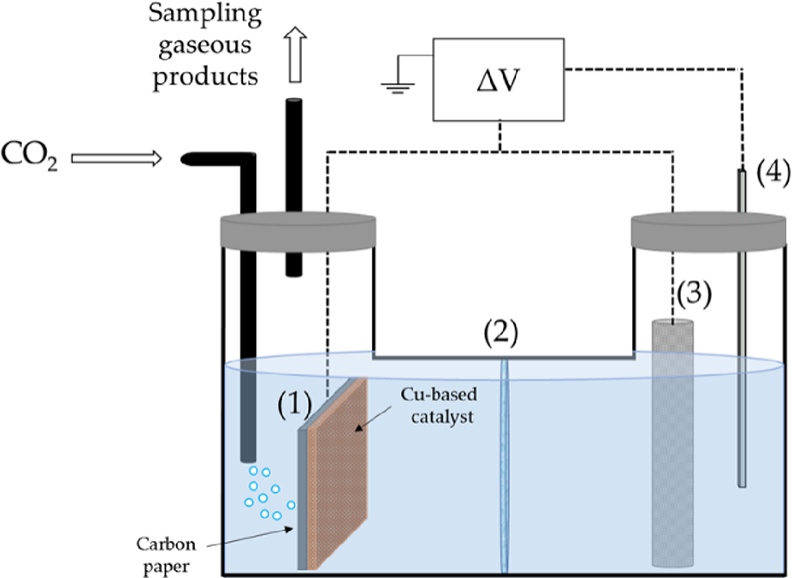
Scheme of the reaction setup for the liquid-phase CO_2_ER. (1) Working electrode, (2) Nafion membrane, (3) counter electrode: Pt gauze, and (4) reference electrode: Ag/AgCl. Electrolyte: 0.3 M KHCO_3_.

The electrochemical reduction tests were performed in an H-type cell equipped with a proton exchange polymeric membrane (Nafion-115). Both sides of the cell were filled with 0.3 M KHCO_3_ as the electrolyte. The cathodic side was presaturated with a constant flux of pure CO_2_ (20 mL min^–1^) for 30 min, which was maintained at 5 mL min^–1^ during the reaction. A Ag/AgCl electrode and a Pt gauze were located in the anodic part of the cell, while the electrocatalyst was placed in the cathodic side (Figure 1). All the applied potentials used for the CO_2_ER are referred to the reversible hydrogen electrode (RHE).

The liquid products were analyzed by a quantitative H^1^ NMR analysis, adding phenol as the internal standard and deuterium oxide to provide an internal lock signal. Gaseous products were collected in a gas sampling bag for all the duration of the electrocatalytic tests and analyzed by gas chromatography. When different reaction times were investigated, a new catalyst was electrodeposited each time.

## 3 Results and Discussion

### 3.1 Carbonaceous Support for the Catalysts

#### 3.1.1 Morphology and XRD Characterization

Toray carbon paper was chosen as the conductive support on which the electrocatalyst was deposited. Mostly known for its large use as a gas diffusion layer (GDL) in the proton exchange membrane fuel cells (PEMFC), the CP structure is composed of three main features.^34^ The long, thin, and randomly distributed fibers provide good electrical conductivity and act as the active phase during electrodeposition, while the void regions among them assure a good carbon dioxide transfer, in both liquid and gas phases. The third feature is ascribed to a carbon binder, which guarantees strength and durability of the fibers. The bare CP was morphologically investigated by SEM (Figure S1a). The majority of the carbon fibers can be individually visualized with an average diameter of 7 μm, but also, fibers linked together by the carbon binder can be distinguished, forming larger areas of conductive fibers. Moreover, X-ray diffraction analysis was conducted in order to evaluate the crystalline structure of the carbon support. The diffraction pattern, which is given in Figure S1b, was compared to the standard ICSD. The reflection pattern was assigned to hexagonal graphite (ICSD no. 98-005-3029),^35^ which shows the most intense signal at 26.5° and another one that is less intense at 54.6°, corresponding to the (002) and (004) planes, according to Miller indices, respectively.

#### 3.1.2 Optimization of the Carbon Gas Diffusion Layer Pretreatment

The cleaning procedure of the catalyst support is a fundamental step to obtain a reproducible and well-adherent coating. In this study, an already reported pretreatment was adopted^36^ that guaranteed (i) an effective cleaning of the carbon support from organic and inorganic impurities without altering the structure of the fibers, (ii) an increase in its hydrophilicity, (iii) good reproducibility of the electrodeposition process, and (iv) short preparation times. H_2_SO_4_ (1 M) and pure EtOH sequential baths were used for this purpose. We tested five treatment combinations in both acidic and alcoholic environments upon variation of the residence times. The effect of the pretreatment was investigated by Raman spectroscopy, and attention was focused on the two typical bands of the carbonaceous materials: the “D band” (1354 cm^–1^), which represents the vibration of the defects inside the crystalline structure, and the “G band” (1581 cm^–1^) that is related to the C–C vibrations of graphite (Figure S2a). The ratio between them, known as the “*R* value”,^37^ indicates the degree of ordered graphite crystallites inside the structure. Figure S2b displays the *R* value (*I*_D_/*I*_G_) related to the five samples coming from the different pretreatments. Overall, the Toray carbon paper after acidic and alcoholic treatments for 2 and 1 h, respectively, reported the lowest ratio (*R* value = 0.19), which means that the disordered carbonaceous components on the surface were reduced, thus favoring a greater contribution of the crystalline structure. Therefore, the aforementioned pretreatment was chosen for the GDL support. Finally, an electrochemically active surface area (ECSA) evaluation was performed using cyclic voltammetry (CV) for the chosen pretreated CP. A potential range of 100 mV centered around the open-circuit value was set, and several CVs with different scan rates were recorded, obtaining an average ECSA of 39 cm^2^ and a roughness factor (RF) of 9.75. The increase in the ECSA in comparison to the geometrical area is fully explained by the fiber structure of the paper.

### 3.2 Copper-Based Catalysts Electrodeposited on the GDL

#### 3.2.1 Synthesis and Morphological/Structural Characterization of the Cu^0^/CP Electrocatalyst

**Figure 2:**
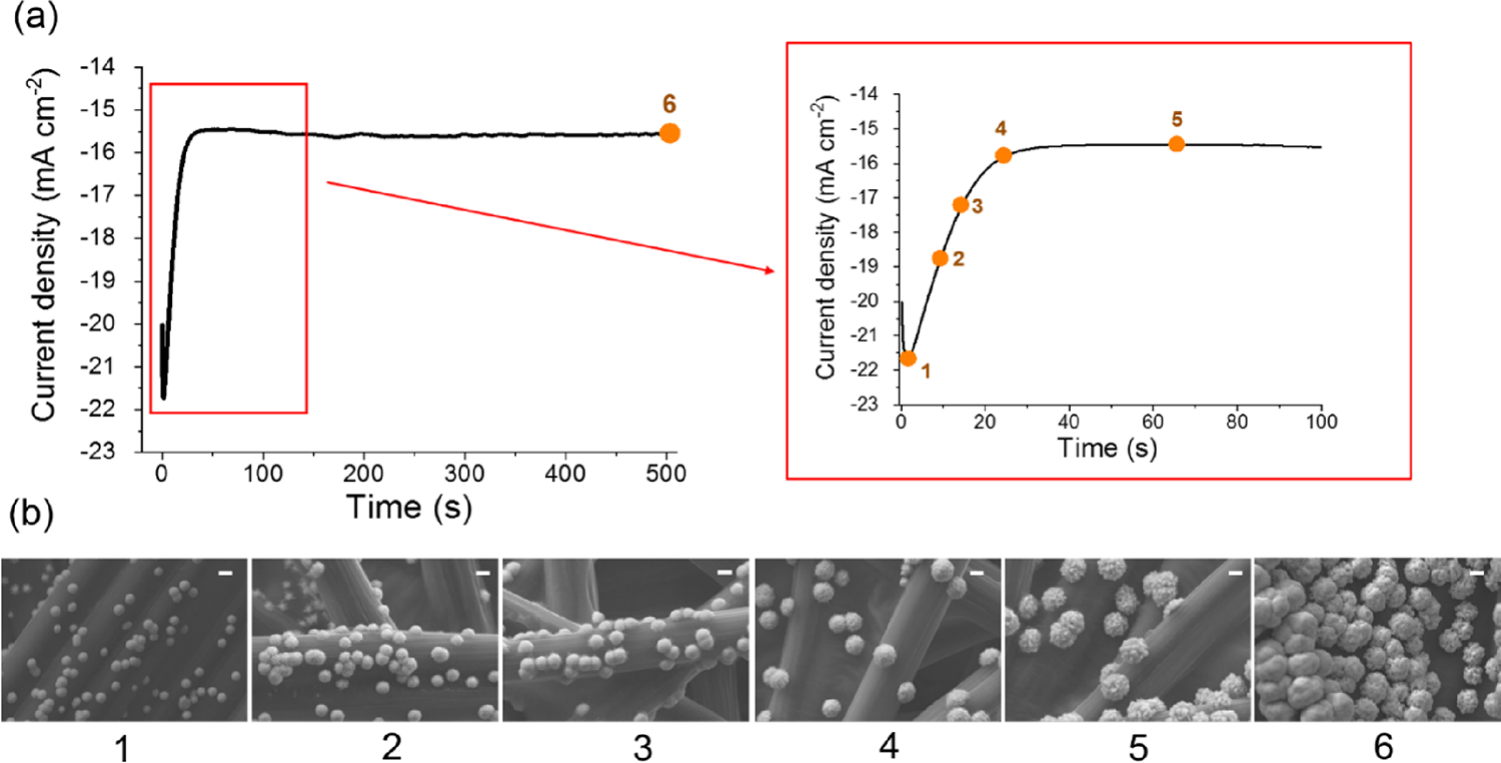
(a) Chronoamperometric curve (*I* vs *t*) recorded during the metal copper deposition over the carbonaceous support of 4 cm^2^ size and (b) SEM images obtained at six different times as shown (scale bar = 1 μm).

The electrodeposition of a thin layer of metal copper over the carbonaceous membrane was performed by applying a constant voltage for a well-defined time, as reported in Figure 2a. The reaction that occurred at the CP, used as the working electrode, is the following
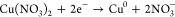


The progress of the reaction was investigated recording SEM images at different times in order to follow the evolution of the Cu^0^ deposition over the carbon fiber. The nucleation of sub-micrometric copper particles started after 1.5 s, as shown in Figure 2b, and a maximum current density of 21.7 mA cm^–2^ was recorded. Afterward, the current density began to decrease and reached a constant value of 15.6 mA cm^–2^ due to the diffusion processes,^38^ while the particles were still growing (Figure S3) until the end of the deposition process.

In addition, as the particles started to increase their dimensions, it can be noticed that crystal agglomerates rather than large single crystals mainly formed.

After 500 s, a homogeneous covering of the conducting fibers was achieved, and the metal copper deposit showed good adhesion to the 3D carbonaceous substrate (Figure S4). Moreover, the reproducibility of the Cu mass was investigated, obtaining an average of 10 ± 1 mg for 20 electrodepositions. Interestingly, the Cu^0^ coating occurred only on the superficial fibers, leaving the underlying ones completely uncovered. Due to the poor interactions of carbon dioxide with the bare fibers, this fact could facilitate the passage of CO_2_ through the 3D structure of the carbon paper to reach the thin catalyst surface oriented toward the anode, where protons are produced. X-ray diffraction spectroscopy was performed on the as-obtained electrocatalyst (Figure S5). The XRD pattern exhibited three distinct reflections ascribable to the Cu^0^ cubic crystal system (ICSD no. 98-006-2848) within the (111), (002), and (022) crystalline planes at 43.6, 50.6, and 74.2°.^39^ Moreover, an average size of 36 nm for the Cu crystallites was achieved by applying the Scherrer’s equation, as given below
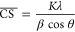
where *K* represents the Scherrer’s constant (0.9), λ is the radiation wavelength, β is the full-width at half-maximum, and θ is the Bragg’s angle.

#### 3.2.2 Electrochemical Characterization of the Cu^0^/CP Electrocatalyst

**Figure 3:**
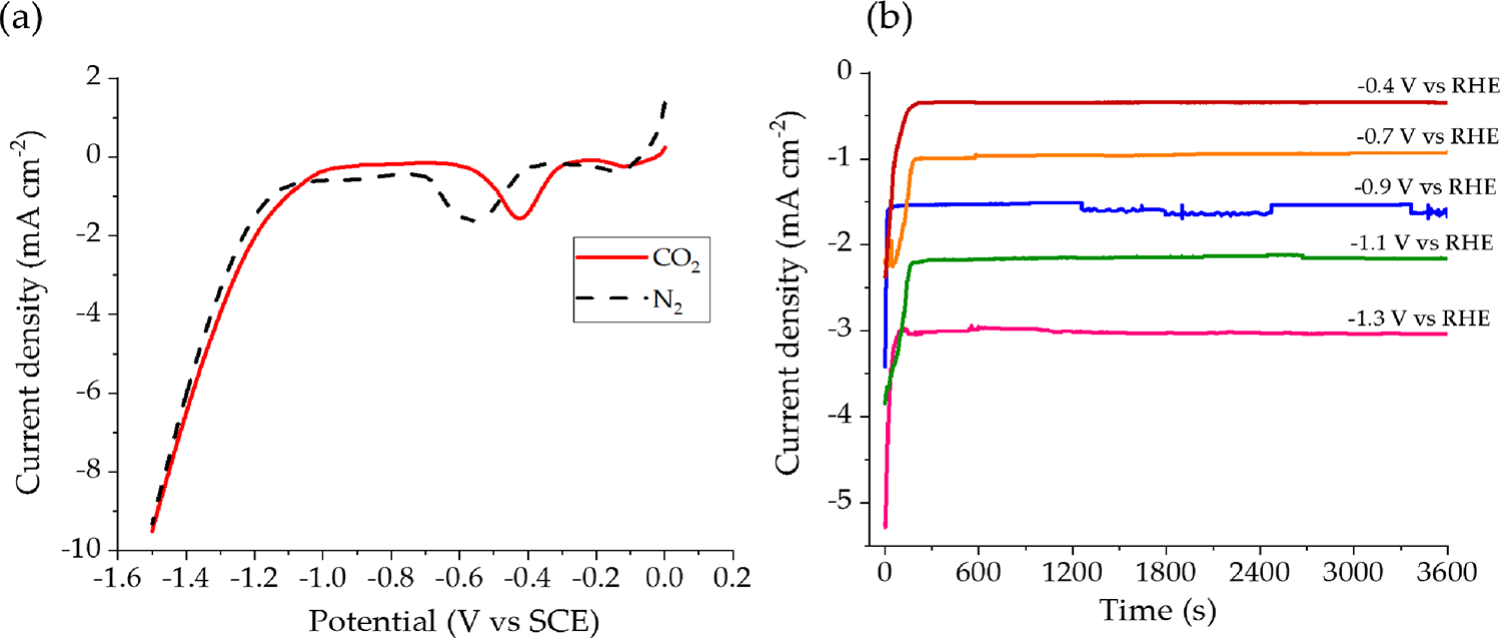
(a) LSV curves of the Cu^0^/CP electrocatalyst in CO_2_ (red line)- and N_2_ (black dashed line)-saturated 0.3 M KHCO_3_. (b) Current densities for CO_2_ER recorded at five Cu^0^/CP electrodes at the shown potentials with CO_2_ bubbling at 5 mL min^–1^.

Figure 3a shows the linear sweep voltammetry (LSV) study carried out on the Cu^0^/CP electrocatalyst, performed in a typical three-electrode cell filled with 0.3 M KHCO_3_, either saturated with N_2_ (inert condition) or CO_2_ (reaction condition). CO_2_ was bubbled for at least 20 min to ensure a constant pH of the electrolyte.^40^ The potential was scanned from 0 to −1.5 V vs SCE, at 10 mV s^–1^.

It can be noticed that the onset potential at which the hydrogen evolution reaction (HER) takes place is slightly anticipated in the presence of CO_2_ (from −1.1 to −0.95 V vs SCE). According to Dongare et al.,^31^ the current density recorded with the N_2_-saturated electrolyte is ascribable to the catalyst electroreduction itself coupled with the hydrogen evolution. On the other hand, the maximum current density recorded after the onset potential shown in the red curve (CO_2_-saturated electrolyte) was slightly higher, probably due to the contribution of the carbon dioxide reduction. The Faradaic peaks observed at −0.42 (red curve) and −0.55 V vs SCE (black curve) can be associated with the reduction of surface Cu oxides locally formed during the LSV characterization. Due to the lower pH of the CO_2_-saturated electrolyte, the reduction of oxidized Cu species was facilitated, occurring at a less cathodic potential. Figure 3b shows several chronoamperometric curves recorded at a Cu^0^/CP electrode during CO_2_ER in different potential-controlled electrolysis conditions with CO_2_ continuously flowing. Herein, the current densities rapidly decreased from the very first step of the reaction until a plateau value, which linearly increased as the applied potential was more cathodic (*R*^2^ = 0.998). Differently from other literature reports,^31^ the potential increase did not lead to significant disturbance in the recorded current.

### 3.3 Oxidative Functionalization and Characterization of the Cu^0^/CP-Derived Electrocatalysts

**Figure 4:**
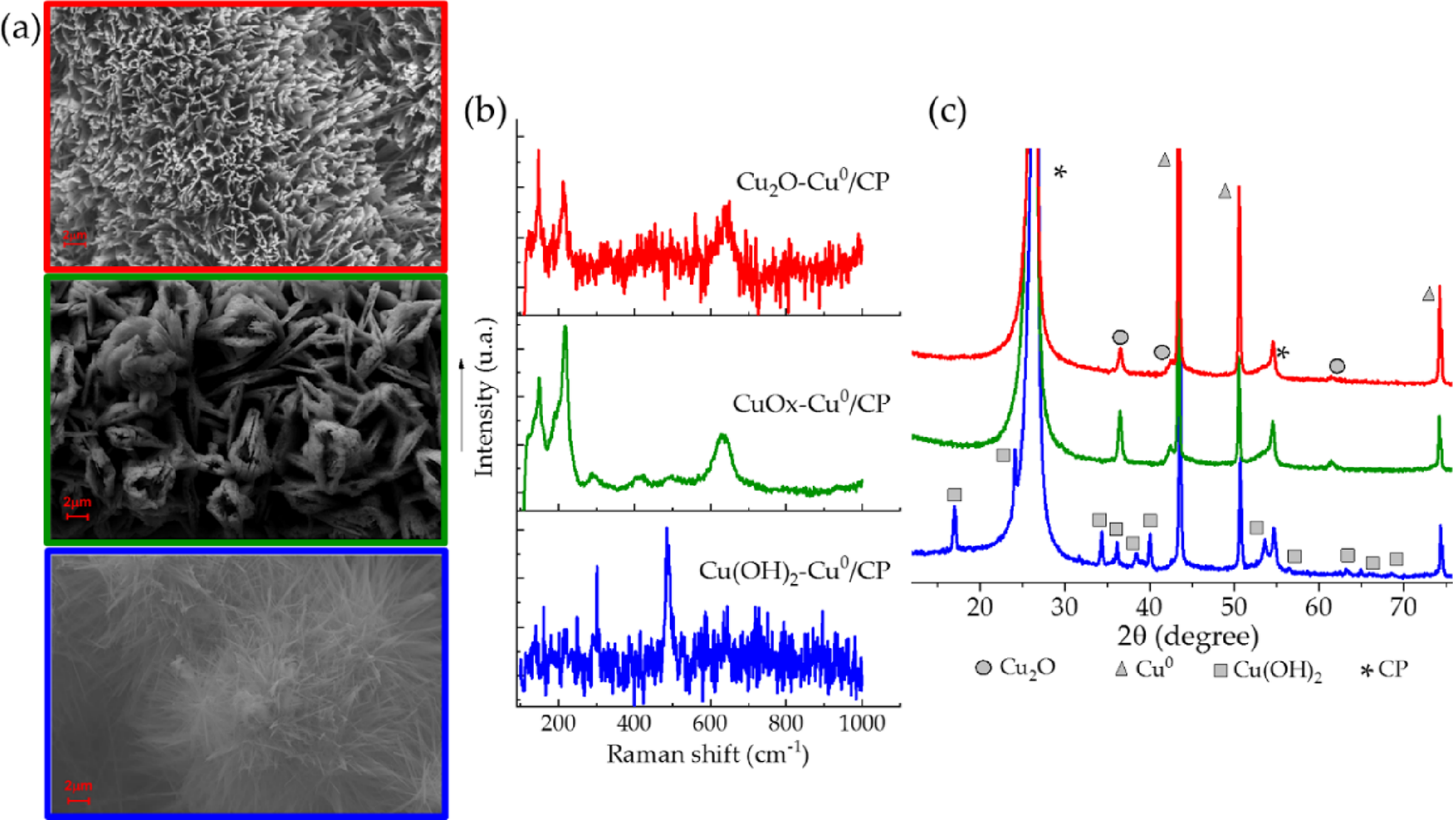
(a) SEM images, (b) Raman spectra, and (c) X-ray diffraction patterns of the oxidized copper species derived from the pristine Cu^0^/CP: Cu_2_O-Cu^0^/CP (red line), CuO_*x*_-Cu^0^/CP (green line), and Cu(OH)_2_-Cu^0^/CP (blue line).

Once the Cu^0^-based gas diffusion electrode was fully investigated, simple chemical and electrochemical methods were employed to selectively change the copper redox couple acting as the active phase. A first functionalization of the Cu^0^ layer was achieved by performing a potential-controlled electrochemical oxidation at −0.4 V vs SCE in 0.5 M KOH as the electrolyte for 12 min, leading to the Cu_2_O-Cu^0^/CP catalyst. The potential for electrochemical oxidation was selected on the basis of a cyclic voltammetry study on the pristine Cu^0^/CP catalyst, as shown in Figure S6, which was in accordance with the literature.^41^ Two further copper oxide-based electrocatalysts were obtained by simple chemical oxidations, soaking the pristine Cu^0^/CP in strong oxidizing mixtures for a relatively short time. The species produced on the pristine metal copper layer were Cu(OH)_2_ and CuO_*x*_, depending on the chemical nature of the oxidant. A comprehensive morphological and structural characterization of the copper-derived samples is reported in Figure 4.

If compared to the metal copper deposit described above, then the SEM images highlight different morphologies, which are typical of the oxidized species, while the corresponding Raman and X-ray diffraction patterns reveal the nature of each catalyst.

In Figure 4, the red-evidenced catalyst was confirmed to be Cu_2_O-Cu^0^/CP by XRD, Raman analyses, and SEM images revealing fibers fully covered by thin and dense flaps with a thickness lower than 100 nm and arranged perpendicularly with respect to the fibers. The electrochemical oxidative treatment has converted the spherical metal copper particles into a segmented nanosheet-like coating characterized by a strong increase in both the surface area of the active material and the void spaces. The X-ray diffraction pattern showed typical reflections at 36.6, 42.5, and 61.7° (ICSD no. 98-006-2467),^42^ referred to the crystalline planes (111), (002), and (022), respectively, likewise of the pristine Cu^0^/CP catalyst crystal structure. Moreover, compared to the crystallite size of the metal copper deposit, the average dimension of the cuprite crystallites was only 3 nm larger, with an average value of around 39 nm. In addition, the Raman spectrum displayed typical peaks of cuprous oxide at 147, 212, and 641 cm^–1^ with much lower intensities than those recorded for the other catalysts,^43^ thus suggesting the presence of a very thin oxidized layer over the pristine metal copper. The catalyst obtained by chemical oxidation in a mixture of 30% v/v H_2_O_2_ and NaOH (Figure 4, green-evidenced) had a uniform shuttle-like microstructure, similarly to the CuO nanoparticles obtained by a sol–gel method developed by Narsinga Rao et al.^44^ Contrary to their nanoparticles, which were 800 nm in length with a thickness of 130 nm, the length of a single shuttle was quite higher at around 8–7 μm with a thickness lower than 600 nm. The Raman analysis displayed the presence of a broad peak at 288 cm^–1^ with a shoulder at 334 cm^–1^, ascribable to the CuO species,^45^ in addition to the typical peaks of Cu_2_O. Moreover, optical microscope images (not shown) revealed the coexistence of weak blue thin fibers growing from the copper deposit along the fibers, similar to the one that will be described just below. Those singular components were hard to analyze because of their smaller sizes, but it was also possible to confirm the presence of Cu(OH)_2_ species (see below). Therefore, by employing a relatively mild chemical oxidation, we were able to obtain an electrocatalyst in which the surface of the metal copper layer was fully covered by all three copper oxide species Cu_2_O, CuO, and Cu(OH)_2_. Due to the small crystallite size of the hydroxide and the bulk nature of the technique, the XRD pattern recorded for this catalyst only highlighted Cu_2_O and Cu^0^ crystalline planes without showing the typical reflections of tenorite species. The average crystallite dimension for Cu_2_O resulted in 28 nm, thus confirming a smaller size than the others. The third catalyst was produced in a stronger oxidizing condition, soaking the Cu^0^/CP in a mixture of ammonium persulfate and sodium hydroxide (blue-evidenced). In this case, the SEM image highlighted the formation of a flower-like structure, where the spherical agglomerates of the metal copper were surrounded by very thin needles, which the further structural characterizations confirmed to be copper hydroxide. In particular, the Raman spectrum exhibited the characteristic peak at 490 cm^–1^,^46^ while the XRD analysis showed a multitude of peaks at 17.0, 24.1, 34.4, 36.2, 38.4, 40.1, 53.6, 56.5, 63.3, 64.9, and 68.62° (ICSD no. 98-002-8484)^47^ referred to the (020), (021), (002), (111), (022), (130), (132), (151), (200), (152), and (221) crystalline planes, respectively, with an average crystallite size of 49 nm.

### 3.4 Liquid-Phase CO_2_ Electroreduction Tests

#### 3.4.1 Performances of the Potentiostatic Electroreduction

The optimized nanostructured electrocatalysts were tested for the reduction of carbon dioxide in liquid phase, as detailed in the Materials section, using the electrochemical setup shown in Figure 1. The pristine Cu^0^/CP catalyst was employed to carry out a complete screening of the potentials applied during a 1 h reaction. The reactions occuring at the electrocatalyst surface are shown in Table 1, together with their standard reduction potentials.

**Table 1:** CO_2_ER Products with Standard Potentials Calculated via the Gibbs Free Energy^a^

reaction	*E*^0^ (V vs RHE)
2H^+^ + 2e^–^ → H_2(g)_	0.0
CO_2_ + 2e^–^ + 2H^+^ → CO_(g)_ + H_2_O	–0.10
CO_2_ + 2e^–^ + 2H^+^ → HCOOH_(aq)_	–0.12
2CO_2_ + 8e^–^ + 8H^+^ → CH_3_COOH_(aq)_ + 2H_2_O	+0.11

a Reported by Zhao et al.^48^

**Figure 5:**
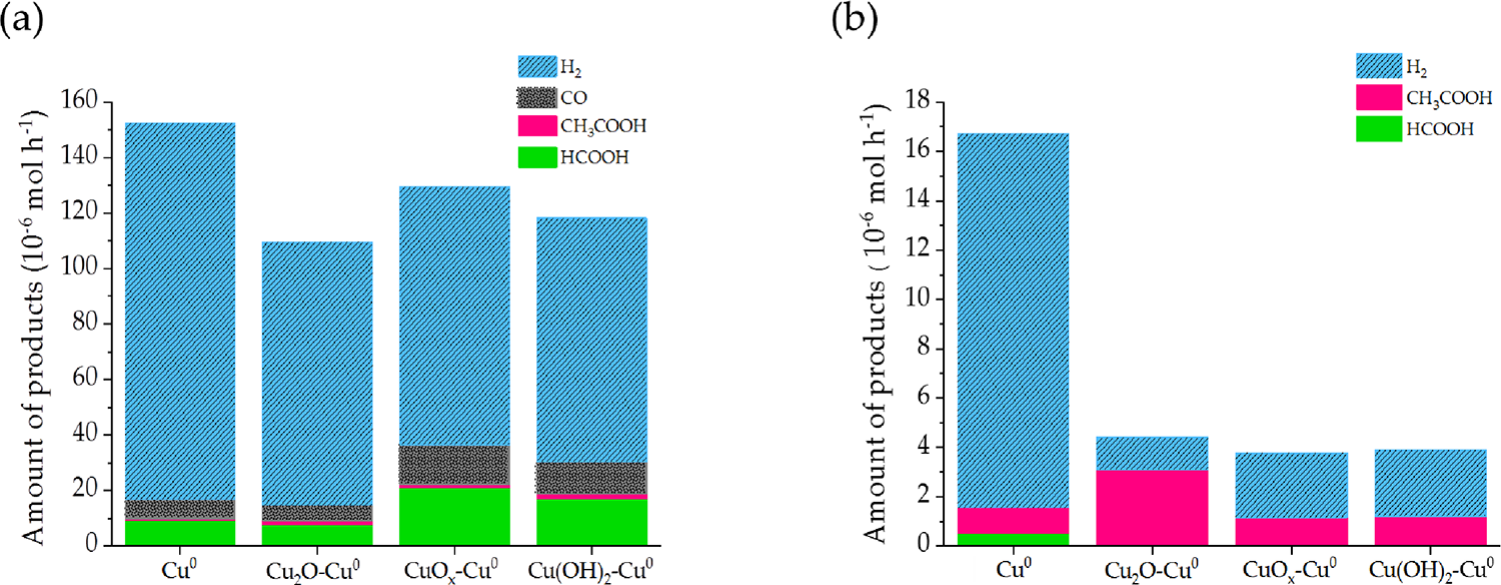
CO_2_ER product distribution for a 1 h reaction at (a) −1.1 V vs RHE and (b) −0.4 V vs RHE.

The product distribution as a function of the applied potentials is reported in Figure S7. It is worth noting that −1.1 V was reported as the potential value at which the highest amount of CO_2_ reduction products can be obtained, as stated by Kuhl et al.,^49^ and, in our case, the best formic acid productivity and selectivity were obtained. Differently, acetic acid was the major product obtained at the least cathodic potential (−0.4 V vs RHE). Moreover, in the latter condition, the lowest amount of H_2_ as a side product was detected. Therefore, these two potentials leading to the most interesting reaction outcomes were investigated during the following tests with the oxidized electrocatalysts. The resulting product distributions are shown in Figure 5, and the recorded current densities are reported in Table S1.

Comparing the results at the two voltages, the first evidence that stands out is the different product distribution. Indeed, by setting the most cathodic potential (−1.1 V vs RHE), hydrogen is observed as the main gaseous product, especially for the pristine Cu^0^/CP electrode, but its presence is due to a side reaction (HER) with no carbon dioxide involved, and a fair amount of carbon monoxide is present. On the other hand, considering the liquid phase, H^1^ NMR analysis revealed the presence of formic acid as the main product and of a small amount of acetic acid. The Faradaic efficiency (FE) was ∼80% for hydrogen evolution and ∼4, 7, and 1% for CO, HCOO^–^, and CH_3_COO^–^ production, respectively.

When using the other three catalysts based on the oxidized species of copper, the same products and an analogous behavior were pointed out, but the FE for the hydrogen evolution decreased to a value of ∼50%, regardless of the employed catalyst.

The greatest amounts of CO (14.4 μmol h^–1^) and HCOOH (21.0 μmol h^–1^) were obtained using the CuO_*x*_-Cu^0^/CP electrocatalyst, and they progressively decreased when the catalysts were Cu(OH)_2_-Cu^0^/CP and Cu_2_O-Cu^0^/CP. Therefore, by applying the most cathodic potential, it was not possible to select the electrocatalyst fulfilling the best compromise between the total moles of products from CO_2_ER and selectivity to useful products (such as acetic and formic acid). Therefore, a lower cathodic potential (−0.4 V vs RHE) was applied. In such a case, the only detected gaseous compound was hydrogen for all the tested catalysts, in much lower amounts with respect to the previous results, and, interestingly, the H^1^ NMR analysis revealed appreciable amounts of acetic acid as the only reduced species in liquid phase.

Again, at the Cu^0^/CP electrode, the highest amount of hydrogen was evolved, so confirming that metal Cu alone does not favor the CO_2_ electroreduction. Indeed, at less cathodic potentials as highlighted in Figure 5b, the selectivity for acetate production was very good, and the electrocatalyst that provided the greatest amount of acetate was the Cu_2_O-Cu^0^/CP. In particular, the produced micromoles were three times higher than the values obtained with the other two electrocatalysts based on oxidized species of copper, and meanwhile, the hydrogen evolution was the lowest. In such a case, the FE was ∼8 and 76% for hydrogen and acetate production, respectively, so confirming the good selectivity of the catalyst for CO_2_ electroreduction to CH_3_COO^–^. Therefore, the Cu_2_O-Cu^0^/CP represented the most active nanostructured catalyst by applying a relatively low cathodic potential, suitable for the application discussed in the Introduction section. Moreover, other literature reports^23,28^ have found out that the copper redox couple Cu^I^/Cu^0^ is the most active one in favoring the C_2_ pathway.

#### 3.4.2 Cu_2_O-Cu^0^/CP versus Cu^0^/CP Productivity

**Figure 6:**
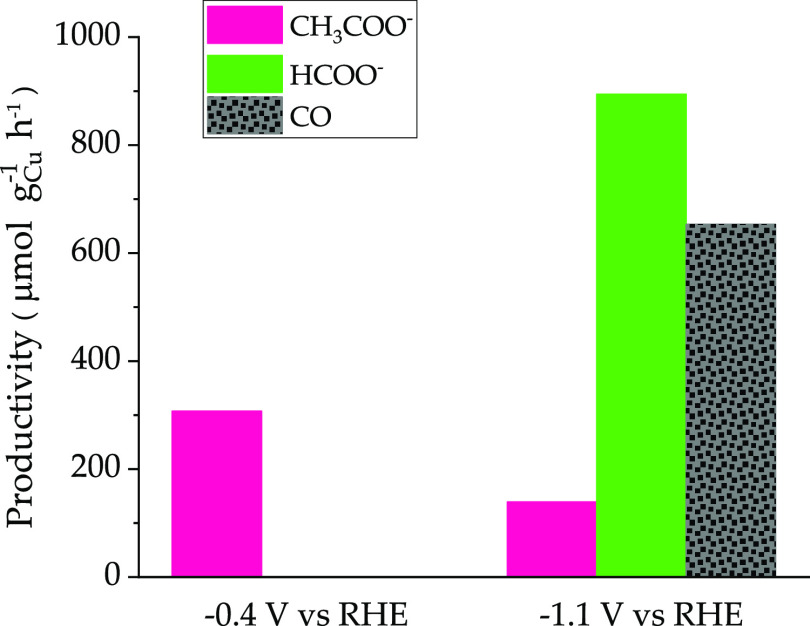
CO_2_ER productivity using the Cu_2_O-Cu^0^/CP electrocatalyst at the two limit reference potentials.

The results obtained with the most promising electrocatalyst at the two limit reference potentials were compared, and the productivity toward the formation of the reduced species was investigated (Figure 6). To this purpose, the micromole production of each compound was referred to the grams of the loaded catalyst (considering the electrodeposited Cu) per hour of reaction. The amount of Cu^0^ deposit on the carbon fibers was calculated by the Faraday’s equation (see the SI). The H_2_ production was not considered in the calculation of the productivity because no carbon dioxide is involved during the occurence of this reaction.

The productivity graph clearly shows the different behavior of the Cu_2_O-Cu^0^/CP electrocatalyst when different cathodic potentials were applied. Interestingly, at −0.4 V vs RHE, the only reduced detected product is acetate with a relatively high productivity of 308 μmol g_cat_^–1^ h^–1^, whereas at −1.1 V, the productivities result in 653, 894, and 139 μmol g_cat_^–1^ h^–1^ for CO, HCOO^–^, and CH_3_COO^–^. All the productivities calculated at the two potentials, when working with Cu_2_O-Cu^0^/CP or Cu^0^/CP electrocatalysts, are collected in Table 2 to emphasize the remarkable change of activity and selectivity passing from the reduced to the partly oxidized state of copper. In particular, at both potentials, the total productivity is higher for the Cu_2_O-Cu^0^/CP electrocatalyst, and the same holds also for the carbon selectivity toward acetate production, resulting in 8.2% (vs 3.1% for Cu^0^/CP) at −1.1 V and practically 100% at −0.4 V. In the following, a tentative explanation of the better performance of the Cu_2_O-Cu^0^/CP than the Cu^0^/CP electrode to produce useful chemicals will be given.

**Table 2:** Productivity (μmol g_cat_^–1^ h^–1^) Obtained for Cu_2_O-Cu^0^/CP and Cu^0^/CP Electrocatalysts at −1.1 and −0.4 V

potential (V vs RHE)	electrocatalyst	CO	HCOOH	CH_3_COOH
–1.1	Cu_2_O-Cu^0^/CP	653	894	139
	Cu^0^/CP	441	590	33
–0.4	Cu_2_O-Cu^0^/CP	nd	nd	308
	Cu^0^/CP	nd	28	56

One of the first works that suggested the key role of the Cu^+^ species in the selective reduction of CO_2_ was published by Mistry et al.,^50^ who developed Cu electrocatalysts using a plasma treatment that produced a stable cuprous oxide layer (Cu_2_O) on the surface of a Cu foil. Also, Roberts et al.^51^ reported a copper surface structured in situ using an oxidative–reductive reaction leading to Cu_2_O, which was subsequently reduced to Cu^0^ in a cubic structure. This catalyst displayed an exceptional ability to catalyze CO_2_ reduction favoring C–C coupling, which leads to multicarbon products.

More recently, Zhu et al.^28^ fabricated a 3D dendritic copper–cuprous oxide composite, which displayed a noticeable selectivity to C_2_ products (acetic acid and ethanol). They attributed the optimum performance of the catalyst to the many exposed active sites in the 3D dendritic structure and to a suitable Cu^I^/Cu^0^ ratio.

Based on these pieces of evidence, the higher activity and the outstanding selectivity of the electrocatalyst Cu_2_O-Cu^0^/CP over Cu^0^/CP can be attributed to the following features, considering that the CO_2_ reduction is a surface process and its efficiency is related to the density of active sites on the catalyst:

(1) One is the proper Cu^I^/Cu^0^ ratio of the catalytic active sites, determined by the electrochemical synthesis above described. In this regard, the coexistence of Cu^I^ and Cu^0^ species at the electrocatalyst surface was predicted to lead to enhanced *CO dimerization and therefore to C_2_ products.^52^
(2) Another is the structure of the Cu_2_O coating, which is preserved also after the application of the cathodic potential at which the CO_2_ER occurs (shown in Figure 4a), constituted of a segmented nanosheet morphology. This structure is characterized by a very high surface area of the active material with respect to the spherical metal copper particles and by many void spaces. The high surface area should guarantee a huge number of active sites produced by the reduction of the Cu_2_O film to Cu^0^, presumably in the form of non-homogeneous nanoparticles with enhanced grain boundaries, which improve the catalytic performance, as already reported.^53^

Overall, the nanosheet-like structure is likely to favor the CO_2_ mass transport throughout the catalyst surface and should guarantee a vast electrochemical interface for CO_2_ activation and reduction. According to the literature, CO_2_ adsorbs on the active sites of the catalyst and is reduced to *CO intermediates that can be more strongly bound on the Cu surface and converted into C_2_ products by *CO dimerization.^31^ That means that *CO dimerization is the key step in the selective reduction of CO_2_ to acetic acid, and this process requires a longer residence time than the one necessary to follow the C_1_ pathway, which produces HCOOH (2H^+^ and 2e^–^).

Moreover, fiber copper electrodes with a three-dimensional porous hollow structure were introduced by Kas et al.^54^ to study the electrochemical reduction of CO_2_, highlighting the importance of the void spaces to remarkably increase the electrocatalytic performance due to the optimal mass transport conditions for those reactions in which at least one gas-phase reactant is involved. The fact that, at the most cathodic potential, a noticeable productivity of CO and HCOOH is observed also at the Cu_2_O-Cu^0^/CP electrode, despite the better selectivity for acetate with respect to the Cu^0^/CP electrode, is thus explainable. At high overpotentials, the current density increases due to the higher rate of both CO_2_ reduction to CO and HER. Consequently, a significant reduction of the CO_2_ concentration at the electrode surface takes place, the reactant is under diffusion control, and the formation of gaseous products limits the formation of multicarbon liquid products, like acetate, at a high concentration.

#### 3.4.3 CH_3_COO^–^ Production over Time Using Cu_2_O-Cu^0^/CP

Although many studies have been carried out to synthesize new materials as catalysts for the electrochemical reduction of CO_2_, in most of the cases, the final products are formate and carbon monoxide, which require further reduction to become useful chemicals. Focusing our attention on acetate, which is the only product apart from hydrogen when the catalyst is Cu_2_O-Cu^0^/CP and the applied potential is −0.4 V vs RHE, electroreductions of different duration (15 min, 30 min, and 1 h, Figure S8) were performed to study the formation kinetics of this product. Table 3 shows the trend of the produced micromoles during the CO_2_ER in 0.3 M KHCO_3_ and continuous CO_2_ feed.

**Table 3:** Acetate Production over Time at −0.4 V vs RHE in 0.3 M KHCO_3_ and Continuous CO_2_ Flow Using Cu_2_O-Cu^0^/CP

time (min)	*J*_tot_ (mA cm^–2^)	CH_3_COO^–^ (μmol)
15	0.60	0.172
30	0.44	0.344
60	0.46	3.09

From the table, it can be noticed that during the first 30 min, the production rate of acetate is constant, whereas in the following 30 min, it increases very rapidly. In Figure S8, the chronoamperometric responses recorded for the three experiments are shown. Looking at Figure S8a, a rapid decrease in the current density recorded at the beginning of the potential application is observed, and such a behavior is due to a reduction of the electrocatalyst itself to covert Cu^I^ into Cu^0^ to obtain a constant ratio of the two oxidation states (activation phase of the electrocatalyst). Immediately after this step, the current stabilizes due to the adsorption/reduction of CO_2_ on the active sites of the catalyst followed by a slow further decrease, related to the mass transport limitation, until a stable value is recorded. Meanwhile, also the hydrogen evolution reaction occurs since the reduction of CO_2_ takes place in aqueous solution, although the applied potential is low and the catalyst displays the lowest activity toward HER with respect to the others investigated in this work. In fact, an evident hydrogen evolution started after ∼200 s, which was visible to the naked eyes. Consequently, electrolysis carried out for shorter times suffered the most from the initial process related to the catalyst activation and the beginning of the HER, thus resulting in a lower acetate production. By applying the potential of −0.4 V for a longer time (1 h), the acetate production remarkably increased during the second half-hour (2.75 vs 0.344 μmol) caused by a higher activity of the Cu^I^/Cu^0^ centers to promote *CO dimerization. However, extending the reaction time to 3 or 5 h, the production of acetate started to decrease. The reasons could be related to a spurious phenomenon due to a possible stripping of the product by the CO_2_ stream or to a poisoning of the catalyst surface induced by cathodic deposition of some metal impurities during the CO_2_ER or by graphitic carbon species formed via decomposition of intermediates.^31,55,56^

#### 3.4.4 Reusability and Stability of the Cu_2_O-Cu^0^/CP Electrode

After 1 h of CO_2_ER at −0.4 V vs RHE, the reusability of the working electrode was also studied. It was disassembled, washed with bidistilled water, dried under nitrogen, and subjected again to the same electrochemical oxidative process to convert Cu to Cu_2_O, as described in the experimental section. Then, it was reused for a second reaction run under the same conditions.

The Faradaic efficiency of acetate during the second CO_2_ER resulted in a slight increase (from 76 to 84%), and the productivity slightly decreased from 308 to 245 μmol g_cat_^–1^ h^–1^. This preliminary experiment, which needs further studies, gives evidence of the possibility to reuse the electrode after a proper activation process, and the Toray carbon paper was not damaged during the disassembling process.

The stability of the optimized catalyst was also investigated over time during a 5 h reaction upon application of −0.4 V vs RHE. The recorded current density plot is shown in Figure S9 and highlights no relevant degradation due to prolonged use, as a relative standard deviation (% RSD) of only 9% on the average *J*_tot_ value was obtained during the whole reaction time.

#### 3.4.5 Comparison of Cu_2_O-Cu^0^/CP Performance with State-of-the-Art Catalysts

In order to compare the performance of the most effective electrocatalyst described in this work, which can reduce CO_2_ to liquid products, a short overview of other Cu-based catalysts reported in the literature is shown in Table 4. It was not easy to get the data since the comparison must involve similar experimental conditions, especially the use of a gas diffusion layer based on carbonaceous paper, the application of a constant potential, and a continuous CO_2_ feed delivered during the liquid-phase reaction. Furthermore, the parameters used in the literature to describe the performance of the catalysts are different and expressed in a different way.

**Table 4:** Overview of Cu-based Electrocatalysts

electrocatalyst	potential (V vs RHE)	electrolyte	products	FE (%)	productivity	reference
porous Cu^0^ NPs	–0.9	0.1 M KHCO_3_	formic acid	40	1460 μM h^–1^	(31)
			acetic acid		10 μM h^–1^	
			ethanol		67 μM h^–1^	
			*n*-propanol		26 μM h^–1^	
dendritic Cu^0^-Cu_2_O composite	–0.7	0.1 M KCl	acetic acid	28.6		(28)
			ethanol	13.2		
Cu^0^ NPs on carbon nanotubes	∼ −1.35	0.5 M KHCO_3_	formic acid	28.5	0.83 μmol h^–1^ (total carbon basis)	(33)
			acetic acid	71.5 (C-based)		
Cu_2_O/GDL	∼ −0.85	0.5 M KHCO_3_	formic acid	8.1	24 μmol h^–1^	(57)
			acetic acid	0.5	0.6 μmol h^–1^	
Cu_2_O-Cu^0^/GDL	–0.4	0.3 M KHCO_3_	acetic acid	76	3.09 μmol h^–1^	this work

Some observations can be done from the data in the table. When the catalyst is based on Cu NPs, the main product is formic acid unless the NPs are supported on carbon nanotubes; on the contrary, if the catalyst contains both Cu^I^ and Cu^0^ centers, then the electrocatalytic reduction activity toward acetic acid is favored but depending on the applied potential. In fact, the major selectivity of the electrochemically produced Cu_2_O-Cu^0^^57^ operating at ∼ −0.85 V vs RHE is toward formate, while with the optimum electrocatalyst proposed in this work, it is possible to obtain only acetic acid with a high FE and an appreciable productivity, working at a low cathodic potential of −0.4 V.

## 4 Conclusions

In this research, affordable and easily synthesized nanostructured Cu-based electrodes with notable catalytic activities toward the electroreduction of carbon dioxide at ambient conditions were reported. The combination of noncritical raw materials and reproducible synthesis/activation procedures, which provides a noteworthy and selective production of added-value chemicals from a waste product, represents a good choice for the application in solar-driven chemistry^2^ and future scalability. Herein, a fully covered 4 cm^2^-sized Cu^0^-based electrocatalyst was successfully obtained by a simple electrochemical deposition on a carbonaceous gas diffusion membrane. Compared to most of the electrocatalysts employed in the state of the art,^7,17,23,28^ the optimized electrode has a higher geometrical surface area, thus overcoming critical steps of reproducibility and providing a scalable approach. Electrochemical and chemical methods are effective strategies to selectively tune the active phase of the pristine catalyst. Therefore, different copper redox couples were obtained by further and simple oxidative functionalization of the pristine Cu^0^/CP, maintaining an average crystallite dimension of less than 40 nm. The catalytic activity of each electrocatalyst toward the liquid-phase electrochemical reduction of CO_2_ was thoroughly investigated at two limit reference potentials. A selectivity toward the formation of acetic acid as the main liquid product was obtained by applying the lowest voltage (−0.4 V vs RHE) at the Cu_2_O-Cu^0^/CP electrocatalyst, thus resulting in the most promising material. Indeed, in this case, the most stable current density was observed that may be due to the decrease in the parasitic hydrogen evolution reaction, in favor of useful product formation. Although the reaction mechanisms at the basis of CO_2_ER are still under debate, we can hypothesize that the use of the Cu^I^/Cu^0^ couple as the active phase preferentially drives the reaction pathway toward C–C bond formation^4,28,51,52^ under mild reaction conditions. In conclusion, the possibility to easily produce nanostructured, large-area, and low-cost electrocatalysts with promising catalytic activities was demonstrated in view of industrially relevant applications.

## Supporting Information Available

SEM image, X-ray diffraction and Raman spectra of the Toray carbon paper surface, SEM analysis of the growth of the metal copper particles, SEM image and X-ray diffraction pattern of Cu^0^ deposition over the carbon fibers, further electrochemical investigation of the Cu^0^/CP electrocatalyst, product distribution, and recorded current densities during CO_2_ER (PDF)
