# Supplementary material for: Nanostructured Copper-Based Electrodes Electrochemically Synthesized on a Carbonaceous Gas Diffusion Membrane with Catalytic Activity for the Electroreduction of CO2
Source: ACS Appl Mater Interfaces. 2021 Nov 26;13(48):57451–61. doi: 10.1021/acsami.1c18844 (PMC8662620; doi:10.1021/acsami.1c18844)
Supplement: Supplementary file 1 — am1c18844_si_001.pdf [file am1c18844_si_001.pdf]

Supporting Information

Nanostructured Copper-based Electrodes Electrochemically Synthesized on a Carbonaceous Gas Diffusion Membrane with Catalytic Activity for the Electroreduction of CO_2

Martina Serafini, Federica Mariani, Andrea Fasolini, Erika Scavetta, Francesco Basile and Domenica Tonelli**

Department of Industrial Chemistry "Toso Montanari", University of Bologna, Viale del Risorgimento, 4, 40136 Bologna, Italy

Corresponding Authors

Domenica Tonelli – *Department of Industrial Chemistry "Toso Montanari", University of Bologna Bologna 40136, Italy;* orcid.org/0000-0002-2844-9817
Email: domenica.tonelli@unibo.it

Francesco Basile – *Department of Industrial Chemistry "Toso Montanari", University of Bologna Bologna 40136, Italy;* orcid.org/0000-0003-1379-6418
Email: f.basile@unibo.it

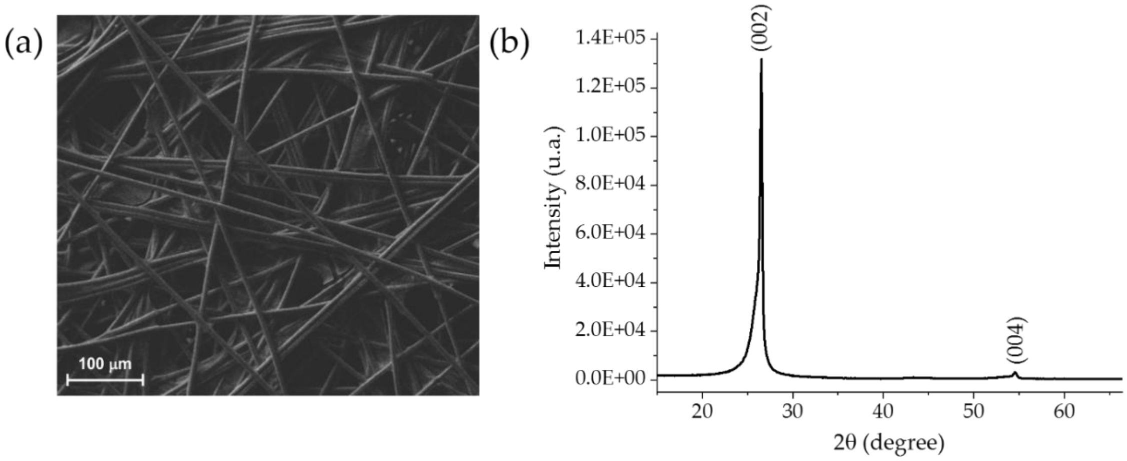

Figure S1. SEM image (a) and XRD diffraction pattern (b) of the pristine Toray carbon paper surface

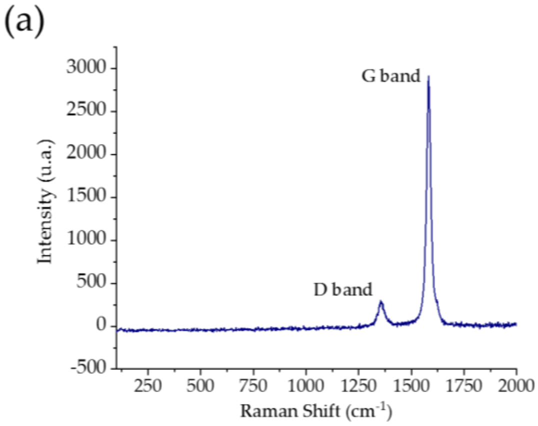

(b)

Pre-treatment method	*R value*
None	0.29
24h 1M H_2SO_4 + 24h pure EtOH	0.24
2h 1M H_2SO_4 + 1h pure EtOH	0.19
8h pure EtOH	0.21
8h 1M H_2SO_4	0.25

Figure S2. (a) Typical D and G bands referred to the disordered and ordered carbonaceous structure and (b) R values obtained for different pre-treatments

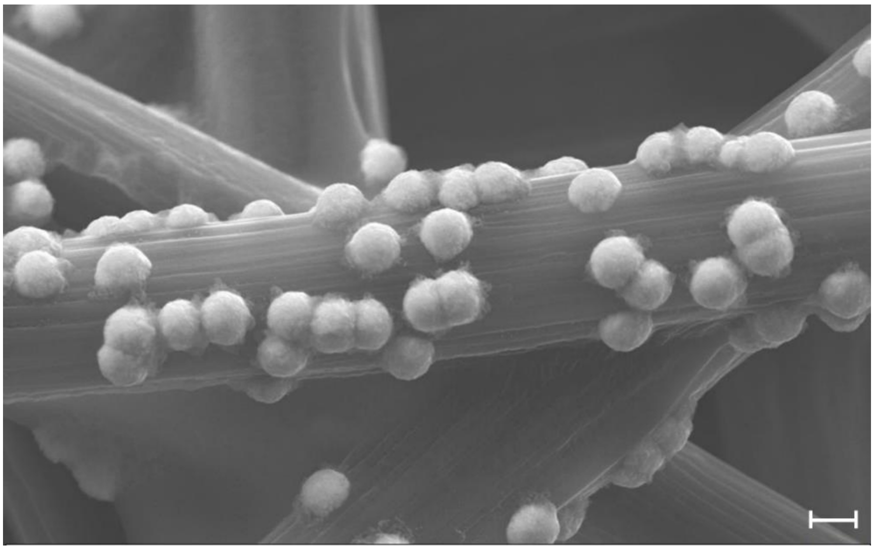

Figure S3. Growth of the metal copper particles over the carbon fiber (17 s passed) Scale bar = 2 µm

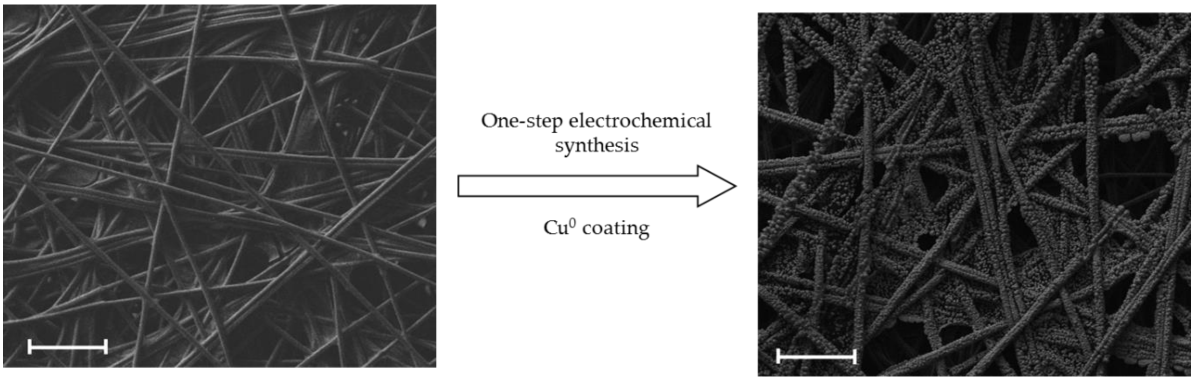

Figure S4. SEM images of the thin metal copper deposition over the carbon fiber support. Scale bar = 100 µm

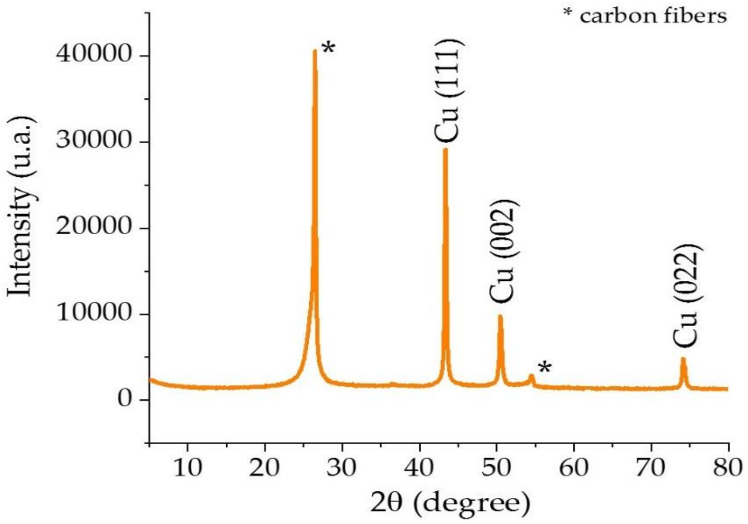

Figure S5 - X-ray diffraction pattern of the copper deposit over the fibers

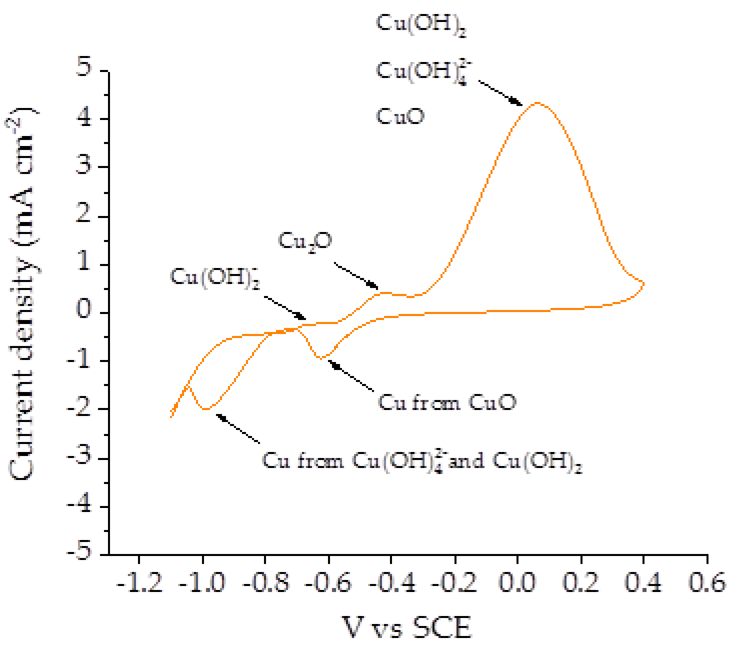

Figure S6. CV study on the Cu^0/CP electrocatalyst in 0.5 M KOH, scan rate 50 mV s^{-1}, N_2 bubbling. Peaks assigned from a reported study [1]

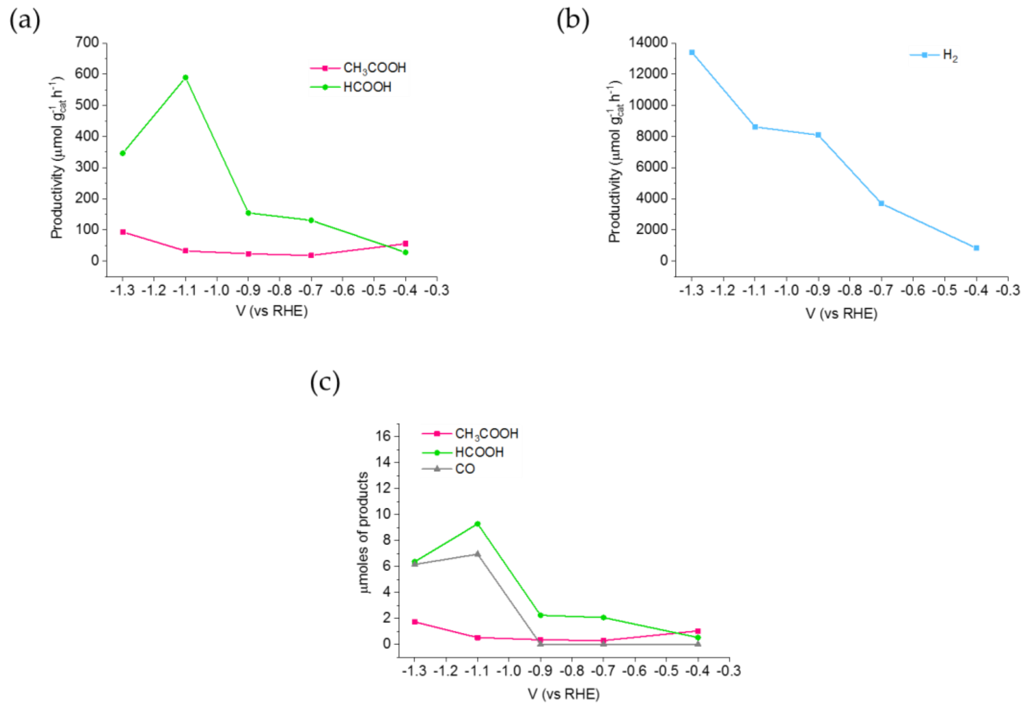

Figure S7. Product distribution obtained during the screening of the applied potentials using the pristine Cu^0/CP electrocatalyst. (a) Productivities of liquid products; (b) productivity of H_2 and (c) detected moles of CO_2ER products

Table S1. Recorded current densities corresponding to the reactions whose product distributions are reported in Figure 5

potential (V vs RHE)	*electrocatalyst*	J_{tot} *(mA cm^{-2})*
**-1.1**	Cu^0	2.20
	Cu_2O-Cu^0	2.58
	CuOx-Cu^0	2.08
	$Cu(OH)_2$-Cu^0	2.32
**-0.4**	Cu^0	0.38
	Cu_2O-Cu^0	0.46
	CuOx-Cu^0	0.93
	$Cu(OH)_2$-Cu^0	0.62

The total amount of copper electrodeposited over the carbonaceous support was calculated by the Faraday's equation:

$$\text{Mass of Cu}^0 = \frac{(\text{AM})\text{Q}}{\text{FZ}}$$

where, AM is the atomic mass of Cu (63.546 g mol^{-1}), Q is the total amount of charge passed during the electrodeposition, F is the Faraday's constant (96485 C mol^{-1}) and Z is the number of the exchanged electrons.

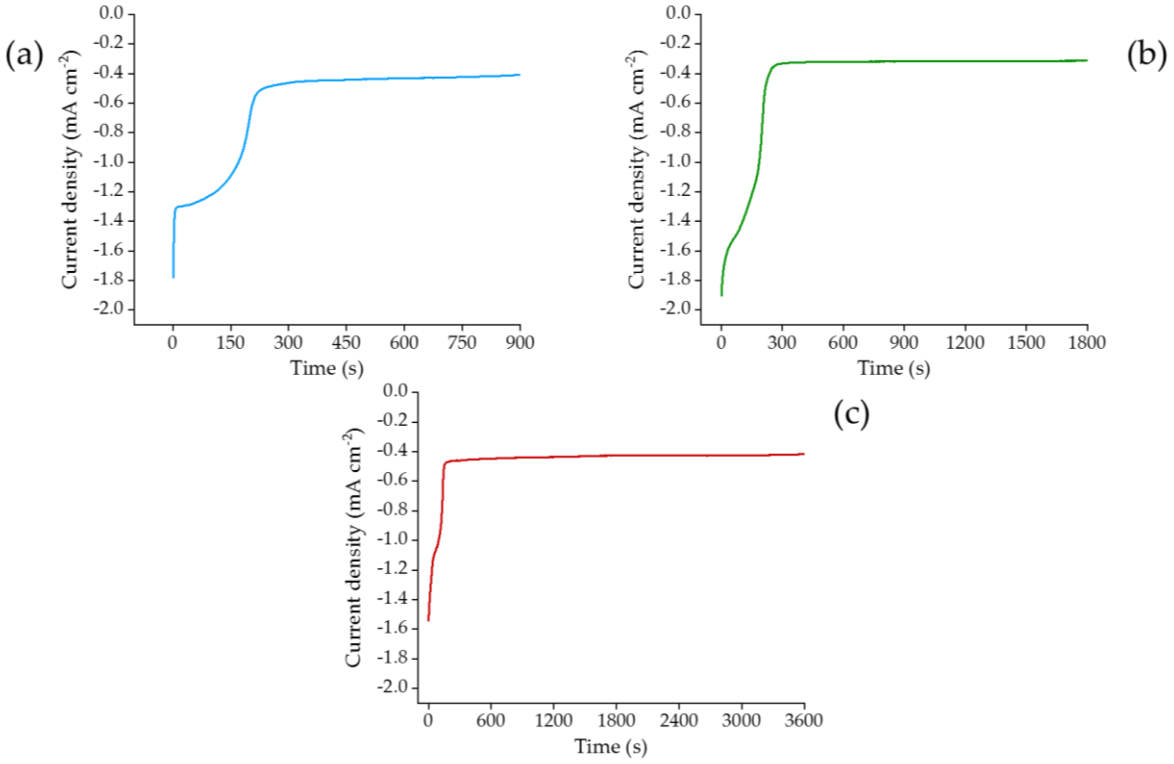

Figure S8. Current densities recorded at Cu_2O-Cu^0/CP electrode during CO_2ER at -0.4 V vs RHE and 0.3 M $KHCO_3$ as electrolyte after (a) 15 min, (b) 30 min and (c) 1h time of reaction

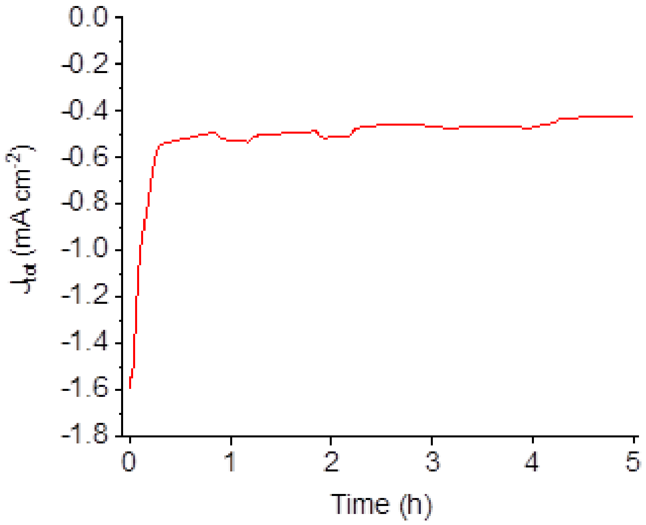

Figure S9. Recorded current density during 5h reaction using Cu_2O-Cu^0/CP at -0.4V vs RHE

References

(1) Giri, S. D.; Sarkar, A. Electrochemical Study of Bulk and Monolayer Copper in Alkaline Solution *J. Electrochem. Soc.* **2016**, *163*, H252-H259. doi.org/10.1149/2.0071605jes
